# Impact of surgery on survival in breast cancer with bone metastases only: a SEER database retrospective analysis

**DOI:** 10.1186/s12893-021-01378-x

**Published:** 2021-10-26

**Authors:** Pai Peng, Jiang-Yuan Chen, Yun-Tao Han, Xin Chen, Hong-Yuan Li, Chao-Hua Hu, Jin-Li Wang

**Affiliations:** 1 Department of Breast and Thyroid Surgery, Xiaogan Hospital Affiliated to Wuhan University of Science and Technology, Square Road No. 6 Xiaogan, Hubei, China; 2 School of Medicine, Jianghan University, 8 Xuefu Road, Wuhan Economic and Technological Development Zone, Wuhan, Hubei China; 3 Department of Breast Surgery, The First Affiliated Hospital of Chongqing Medical University, 1 Youyi Road, Yuanjiagang, Yuzhong, Chongqing, China; 4 Department of Breast Surgery, Jingzhou Central Hospital, No. 60 Jingjing Road, Jingzhou, Jingzhou, Hubei China

**Keywords:** Breast cancer, Bone metastases, SEER database, Primary surgery, Prognosis, Overall survival

## Abstract

**Background:**

It was controversial to operate on the primary site of breast cancer (BC) with bone metastasis only. We investigated the impact of surgery on BC patients with bone metastases via a SEER database retrospective analysis.

**Methods:**

A total of 2917 BC cases with bone metastasis, first diagnosed between 2010 and 2015 in the Surveillance, Epidemiology, and Results Database (SEER) of National Cancer Institute were selected. We assessed the effect of different surgical procedures on survival and prognosis.

**Results:**

Compared with the non-surgical group, the primary tumor surgical group showed longer median survival time (χ^2^ = 146.023, P < 0.001), and the breast-conserving subgroup showed the highest median survival time of 70 months (χ^2^ = 157.117, P < 0.001). Compared with the non-surgery group, the median overall survival (OS) of primary surgery group was longer (HR = 0.525, 95%CI = 0.467–0.590, P < 0.001), and the breast-conserving subgroup showed the longest median operative OS (HR = 0.394, 95%CI = 0.325–0.478, P < 0.001).

**Conclusion:**

This study showed that primary surgery could improve the median survival time and OS of BC patients with bone metastasis. Moreover, under the condition of low tumor burden, breast conserving surgery was a better choice.

## Background

Breast cancer (BC) is the most common malignant tumor threatening women's health, with 270,000 newly diagnosed BC patients yearly and approximately 42,000 related deaths, which ranks first in morbidity and second in mortality worldwide [1]. With the improvement of people's health awareness and the development of medical technology, the screening and treatment of early BC have been significantly improved. However, about 5% of BC patients had bone metastasis at the first diagnosis, and the incidence of bone metastasis was 70% in advanced metastatic BC [2, 3]. Currently, the treatment principle for BC patients with bone metastasis is to relieve pain, restore function and improve the quality of life, increase the survival time, and the main comprehensive therapy includes radiation and chemotherapy [4–6].

Accumulating evidence indicates that the active primary site surgery was associated with survival benefits in many retrospective studies [7–15], although few prospective studies produced positive results [16–18]. Routine screening of bone metastases in patients with localized BC was not recommended by the current guideline only if signs or symptoms occurred. The effect of operation on the primary site of BC with bone metastasis is still controversial.

In this study, we used the SEER database to investigate the effect of surgical operation on the prognosis of BC patients with bone metastases from 2010 to 2015.

## Methods

### Data

We obtained data from SEER Medicare database, which consists of 18 population-based cancer registries. The SEER program of the National Cancer Institute collects and publishes cancer incidence and survival data encompassing approximately 28% of the United States population. In this study, we used SEER*Stat Version 8.3.6 (http://www.seer.cancer.gov/seerstat) from the National Cancer Institute to survey eligible patients.

### Patients

We collected BC patients with bone metastases between 2010 and 2015 based on the 7th edition of the American Joint Committee on Cancer (AJCC) Cancer Staging Manual [19]. We further screened the patients through inclusion criteria and exclusion criteria, inclusion criteria: (1) Pathological diagnosis of breast cancer; (2) Age > 18 years old female; (3) Simple distant bone metastases occurred clearly at the first diagnosis; (4) Complete clinicopathological information such as tumor pathological type and histological grade; (5) Complete treatment information; (6) Prognostic information is complete. Exclusion criteria: (1) Multiple primary carcinomas; (2) Bilateral breast cancer; (3) Patients with unknown T and N stages, such as T0,TX and NX, were excluded. After relevant screening, we finally leaved 2917 patients eligible for survival analyses and related research.

### Clinicopathological parameters and control variables

Patients were divided into primary surgery group and non-surgery group. The Surgery group was divided into Breast Conservion Surgery (BCS) group, subcutaneous or simple Mastectomy group (Mastectomy), modified Radical Mastectomy group and Radical Mastectomy group (Radical mastectomy). Clinical pathology staging was based on AJCC 7th edition. The following clinicopathological characteristics were used as study factors: age, race, ethnic origin, marital status, T stage, N stage, laterality, behavior, ER, PR, HER2, tumor subtype and histological grade, radiotherapy, and chemotherapy.

### Statistical analysis

Patient and tumor characteristics were summarized using descriptive statistics and compared using a two-sided χ^2^ test for categorical variables and Student’s t test for continuous variables. In the survival analysis, Kaplan–Meier method was used to calculate the median survival time, and log-rank test was used for univariate analysis. Statistically significant factors in univariate analysis results were incorporated into COX proportional hazard regression model for multivariate analysis. All the above statistical analyses were calculated by SPSS 22.0 statistical software. Two-sided P values < 0.05 were considered statistically significant.

## Results

### Patient characteristics

A total of 2917 BC patients with bone metastasis only were included in this study, and clinical characteristics were listed in Table 1. The primary surgery group included 1245 cases (42.7%, 1235/2917), and the non-surgery group consisted of 1672 cases (57.3%, 1672/2917). The clinicopathological parameters showed significant differences between two groups included marital status, age, histological grade, T stage, N stage, ER, molecular subtype, radiotherapy and chemotherapy. Compared with the non-operative patients, the proportion of young patients undergoing surgery was higher (χ^2^ = 21.613, P < 0.001). Compared with unmarried patients, married patients had a higher surgical rate (46.8% vs 38.8%). Patients in advanced stage had more ratio of radiotherapy and chemotherapy. Compared with ER positive, ER + /HER2− and ER + /HER2 + patients, ER negative, TNBC, and HER2-enriched BC patients had higher surgical rate (P < 0.001 and P = 0.009). There were no significant differences in race, Ethnic origin, behavior, laterality, HER2 and PR expression between the two groups.

**Table 1:** The baseline level of 2917 patients

Variables	Number	Surgery (%)	Non-surgery (%)	χ2	P-value
*Race*					
White	2255	968 (42.9%)	1287 (57.1%)	0.567	0.753
Black	462	190 (41.1%)	272 (58.9%)		
Other	200	87 (43.5%)	113 (56.5%)		
*Ethnic origin*					
Spanish-Hispanic-Latino	289	136 (47.1%)	153 (52.9%)	2.513	0.113
Non-Spanish-Hispanic-Latino	2628	1109 (42.2%)	1519 (57.3%)		
*Age*					
≤ 35	134	67 (50%)	67 (50%)	21.613	< 0.001
35 < Age < 60	1345	631 (46%)	714 (54%)		
≥ 60	1438	547 (38%)	891 (62)		
*Marital status*					
Married	1416	662 (46.8%)	754 (53.2%)	18.638	< 0.001
Unmarried	1501	583 (38.8%)	918 (61.2%)		
*Grade*					
1	287	99 (34.5%)	188 (65.5%)	37.276	< 0.001
2	1440	560 (38.9%)	880 (68.1%)		
3–4	1190	586 (49.2%)	604 (50.8%)		
*Laterality*					
Left	1525	656 (43%)	869 (57%)	0.147	0.701
Right	1392	589 (42.3%)	803 (57.7%)		
*Histology*					
IDC	2351	1023 (43.5%)	1328 (56.5%)	3.433	0.064
Non-IDC	566	222 (39.2%)	344 (60.8%)		
*T stage*					
T1	383	153 (39.9%)	230 (60.1%)	45.68	< 0.001
T2	1158	569 (49.1%)	589 (50.9%)		
T3	553	242 (43.8%)	311 (56.2%)		
T4	823	281 (34.1%)	542 (65.9%)		
*N stage*					
N0	704	208 (29.5%)	496 (70.5%)	258.366	< 0.001
N1	1326	463 (34.9%)	863 (65.1%)		
N2	406	261 (64.3%)	145 (35.7)		
N3	481	313 (65.1%)	168 (34.9%)		
*Radiation*					
Yes	1302	704 (54.1%)	598 (45.9%)	124.703	< 0.001
No	1615	541 (33.5%)	1074 (66.5%)		
*Chemotherapy*					
Yes	1564	803 (51.3%)	761 (48.7%)	103.411	< 0.001
No	1353	442 (32.7%)	911 (67.3%)		
*Subtype*					
ER + /HER2−	2114	869 (41.1%)	1245 (58.9%)	11.671	0.009
ER + /HER2 +	433	189 (43.6%)	244 (56.4%)		
HER2 +	122	61 (50%)	61 (50%)		
TNBC	248	126 (50.8%)	122 (49.2%)		
*ER*					
Positive	2532	1050 (41.5%)	1482 (58.5%)	11.512	0.001
Negative	385	195 (50.6%)	190 (49.4%)		
*PR*					
Positive	2121	886 (41.8%)	1235 (58.2%)	2.62	0.106
Negative	796	359 (45.1%)	437 (54.9%)		
*HER2*					
Positive	555	250 (45%)	305 (55%)	1.566	0.211
Negative	2362	995 (42.1%)	1367 (57.9%)		

*ER* estrogen receptor, *PR* progesterone receptor, *Her-2* human epidermal growth factor receptor 2, *IDC* infiltrating ductal carcinoma, *TNBC* triple-negative breast cancer

### Univariate analysis and multivariate analysis on the prognosis of BC with bone metastasis

**Fig. 1:**
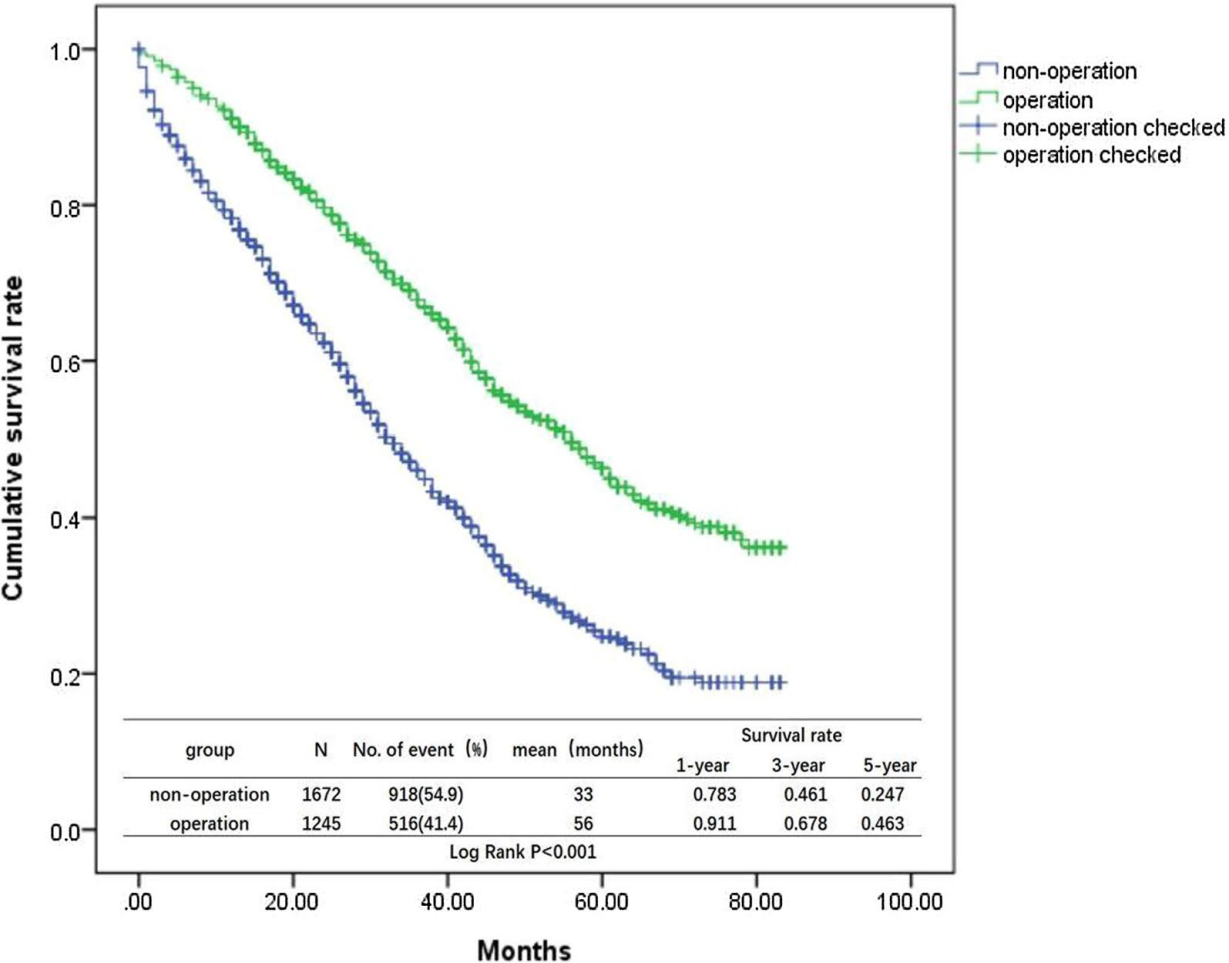
Survival curves for non-operation vs operation

**Fig. 2:**
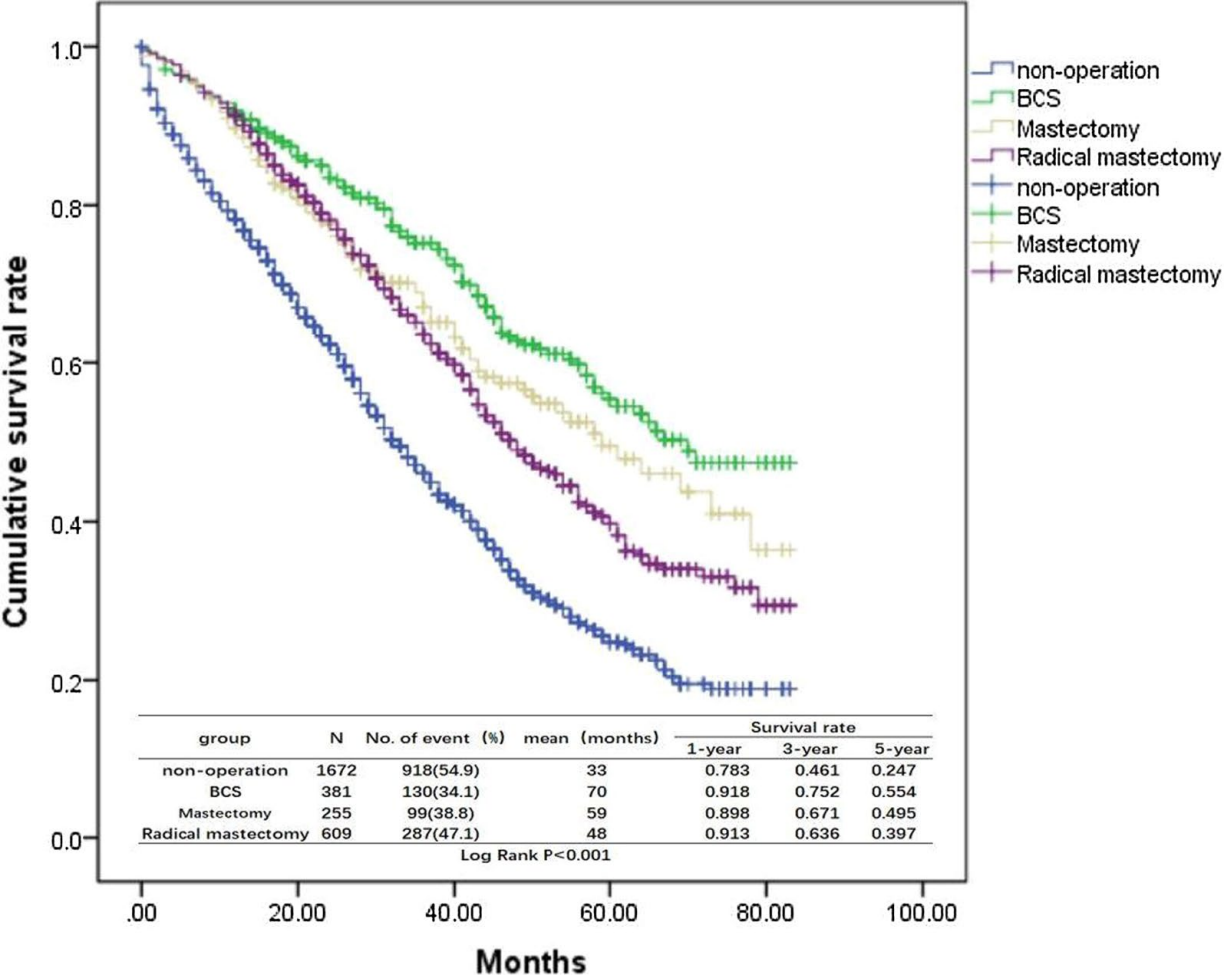
Survival curves for non-operation vs different surgical modes

Univariate analysis was performed for the 2917 cases (Table 2), and the clinicopathological parameters influencing the prognosis of patients were race, histologic grade, marital status, age, histology, T stage, tumor radiotherapy and chemotherapy, ER, PR, and HER2, subtypes, primary tumors surgery and surgical procedure (P< 0.05). Ethnic origin, laterality, and N staging showed no significant difference (P > 0.05). According to the results of univariate analysis, primary site surgical group significantly affected the prognosis of patients (Fig. 1) compared to the non-surgical group (χ^2^ = 146.023, P<0.001); Among the surgery group, the survival time of the breast-conserving group (Fig. 2) benefitted most for 70 months (χ^2^ = 157.117, P < 0.001). Advanced stage patients showed decreased median survival time (P < 0.001). Compared with the black, the white and others got a longer survival time (χ^2^ = 35.071, P < 0.001); The expression of ER, PR, and HER2, histology of invasive ductal carcinoma, married patients, younger age, radiotherapy, and chemotherapy were all protective factors for BC with bone metastasis (P < 0.001). Univariate analysis showed that her2-positive BC had the best prognosis, with the median survival time up to 73 months, while TNBC had the worst prognosis, with the median survival time of 13 months. COX multivariate regression analysis was performed based on the pathological parameters screened by univariate analysis (Table 3). The results showed that the clinicopathological parameters with significant differences between the two groups included race, age, marital status, histological grade, histology, chemotherapy, ER, PR, HER2, molecular subtype, T stage, primary surgery and surgical mode. The results of univariate analysis were consistent with COX multivariate regression analysis except the slight change of radiotherapy factors.

**Table 2:** Univariate analysis of clinical pathology and prognosis of 2917 BC patients with bone metastasis

Variables	Number	Median survival time (month)	95% confidence interval (CI)	χ2	P-value
*Race*					
White	2255	44	41.617–46.383	35.071	< 0.001
Black	462	31	27.607–34.393		
Other	200	43	31.973–54.027		
*Grade*					
1	287	46	37.721–54.279		
2	1440	46	42.998–49.002	45.128	< 0.001
3–4	1190	34	31.198–37.002		
*Marital*					
Married	1416	48	44.517–51.483	45.771	< 0.001
Unmarried	1501	36	33.209–38.791		
*Age*					
≤ 35	134	56	38.548–73.452		
35 < age < 60	1345	46	42.387–49.613	70.468	< 0.001
≥ 60	1438	36	33.066–38.934		
*Ethnic origin*					
Spanish-Hispanic-Latino	289	47	41.597–52.403	2.103	0.147
Non-Spanish-Hispanic-Latino	2628	41	38.983–43.017		
*Laterality*					
Left	1525	42	39.446–44.554	0.017	0.896
Right	1392	42	39.085–44.915		
*Histology*					
IDC	2351	44	41.537–46.463	25.295	< 0.001
Non-IDC	566	36	32.245–39.755		
*T stage*					
T1	383	47	38.824–55.176		
T2	1158	47	42.855–51.145	53.468	< 0.001
T3	553	40	35.692–44.308		
T4	823	34	30.984–37.016		
*N stage*					
N0	704	39	34.668–43.332		
N1	1326	43	39.941–46.059	5.582	0.134
N2	406	43	37.639–48.361		
N3	481	42	38.004–45.996		
*Radiation*					
Yes	1302	46	42.593–49.407	14.906	< 0.001
No	1615	39	36.512–41.488		
*Chemotherapy*					
Yes	1564	48	44.242–51.758	64.579	< 0.001
No	1353	36	33.254–38.746		
*Subtype*					
ER + /HER2−	2114	43	41.030–44.970		
ER + /HER2 +	433	57	48.725–65.275	242.199	< 0.001
HER2 +	122	73	0		
TNBC	248	13	11.349–14.651		
*ER*					
Positive	2532	44	42.077–45.923	91.338	< 0.001
Negative	385	18	14.319–21.681		
*PR*					
Positive	2121	46	43.765–48.235	97.923	< 0.001
Negative	796	27	23.973–30.027		
*HER2*					
Positive	555	58	46.618–69.382	30.843	< 0.001
Negative	2362	40	37.931–42.069		
*Primary surgery*					
Operation	1245	56	51.508–60.492	146.023	< 0.001
Non-operation	1672	33	30.830–35.170		
*Surgical mode*					
Non-operation	1672	33	30.830–35.170		
BCS	381	70	0	157.117	< 0.001
Mastectomy	255	59	45.751–72.249		
Radical mastectomy	609	48	43.369–52.631		

*ER* estrogen receptor, *PR* progesterone receptor, *Her-2* human epidermal growth factor receptor 2, *IDC* infiltrating ductal carcinoma, *BCS* breast conserving surgery, *TNBC* triple-negative breast cancer

**Table 3:** Multivariate analysis of clinical pathology and prognosis of 2917 BC patients with bone metastasis

Variables	Regression coefficient	Standard error	P-value	HR	95%CI
*Marital*					
Married	Reference				
Unmarried	0.193	0.056	0.001	1.213	1.087–1.354
*Age*					
≤ 35	Reference				
35 < age < 60	− 0.07	0.147	0.635	0.933	0.700–1.243
≥ 60	0.258	0.147	0.08	1.294	0.970–1.727
*Race*					
White	Reference				
Black	0.267	0.071	< 0.001	1.306	1.135–1.502
Other	0.001	0.113	0.995	1.001	0.802–1.248
*Histology*					
IDC	Reference				
Non-IDC	0.331	0.065	< 0.001	1.392	1.225–1.583
*Primary surgery*					
Non-operation	Reference				
Operation	− 0.644	0.059	< 0.001	0.525	0.467–0.590
*Chemotherapy*					
Yes	Reference				
No	0.383	0.061	< 0.001	1.467	1.302–1.653
*T stage*					
T1	Reference				
T2	0.116	0.091	0.2	1.123	0.940–1.341
T3	0.247	0.099	0.013	1.28	1.054–1.555
T4	0.321	0.093	0.001	1.379	1.149–1.654
*Radiation*					
Yes	Reference				
No	− 0.011	0.055	0.849	0.99	0.888–1.103
*Grade*					
1	Reference				
2	0.234	0.1	0.02	1.263	1.038–1.538
3–4	0.583	0.105	< 0.001	1.791	1.459–2.199
*HER2*					
Positive	Reference				
Negative	0.571	0.082	< 0.001	1.771	1.508–2.079
*ER*					
Positive	Reference				
Negative	0.589	0.095	< 0.001	1.802	1.496–2.170
*PR*					
Positive	reference				
Negative	0.442	0.073	< 0.001	1.556	1.348–1.797
*Subtype*					
ER + /HER2−	Reference				
ER + /HER2 +	− 0.243	0.091	0.007	0.785	0.657–0.937
HER2 +	− 2.024	0.353	< 0.001	0.132	0.066–0.264
TNBC	− 0.547	0.322	0.089	0.579	0.308–1.088
*Surgical mode*					
Non-operation	Reference				
BCS	− 0.93	0.099	< 0.001	0.394	0.325–0.478
Mastectomy	− 0.702	0.108	< 0.001	0.496	0.401–0.613
Radical mastectomy	− 0.488	0.071	< 0.001	0.614	0.534–0.706

*ER* estrogen receptor, *PR* progesterone receptor, *Her-2* human epidermal growth factor receptor 2, *IDC* infiltrating ductal carcinoma, *BCS* breast conserving surgery, *TNBC* triple-negative breast cancer

### Comparison of baseline data among 1245 patients with different surgical modes

To further analyze the difference in survival analysis among different surgical methods, we used χ^2^ test to further analyze the baseline data of 1245 patients with different surgical methods (Table 4). T staging, N staging, radiotherapy and chemotherapy showed significant differences (P < 0.05) in baseline data of 1245 patients, while no significant differences in other variables were found. In the BCS group, the proportion of T and N stages, radiotherapy and chemotherapy was higher: the clinical stages were lower, the tumor burden was lower, and the treatment was more active.

**Table 4:** Baseline data analysis of 1245 patients with different surgical mode

Variables	BCS (%)	Mastectomy (%)	Radical Mastectomy (%)	χ^2^	P-value
*Race*					
White	297 (78.0%)	204 (80.0%)	467 (76.7%)		
Black	57 (15.0%)	38 (14.9%)	95 (15.6%)	2.12	0.714
Other	27 (7.0%)	13 (5.1%)	47 (7.7%)		
*Age*					
≤ 35	13 (3.4%)	16 (6.3%)	38 (6.2%)		
35 < age < 60	181 (47.5%)	132 (51.8%)	318 (52.2%)	8.373	0.079
≥ 60	187 (49.1%)	107 (42.0%)	253 (41.5%)		
*Grade*					
1	34 (8.9%)	20 (7.8%)	45 (7.4%)	6.528	0.163
2	173 (45.4%)	129 (50.6%)	258 (42.4%)		
3–4	174 (45.7%)	106 (41.6%)	306 (50.2%)		
*Laterality*					
Left	209 (54.9%)	130 (51.0%)	317 (52.1%)	1.115	0.573
Right	172 (45.1%)	125 (49.0%)	292 (48.0%)		
*Marital*					
Married	206 (54.1%)	138 (54.1%)	318 (52.2%)		
Unmarried	175 (45.9%)	117 (45.9%)	291 (47.8%)	0.438	0.803
*Ethnic origin*					
Spanish-Hispanic-Latino	43 (11.3%)	23 (9.0%)	70 (11.5%)	1.205	0.547
Non-Spanish-Hispanic-Latino	338 (88.7%)	232 (91.0%)	539 (88.5%)		
*Histology*					
IDC	326 (85.6%)	201 (80.0%)	49 (77.0%)	5.163	0.076
Non-IDC	55 (14.4%)	54 (20.0%)	113 (33.0%)		
*T stage*					
T1	91 (23.9%)	21 (8.2%)	41 (6.7%)	158,845	< 0.001
T2	22 (58.3%)	109 (42.7%)	238 (39.10%)		
T3	37 (9.7%)	59 (23.1%)	146 (24%)		
T4	31 (8.1%)	66 (25.9%)	184 (30.20%)		
*N stage*					
N0	126 (33.1%)	52 (20.4%)	30 (4.9%)		
N1	133 (34.9%)	124 (48.6%)	206 (33.80%)	192.317	< 0.001
N2	67 (17.6%)	41 (16.10%)	153 (25.10%)		
N3	55 (14.4%)	38 (14.90%)	220 (36.10%)		
*Radiation*					
Yes	22 (60.1%)	124 (48.6%)	351 (57.60%)		
No	15 (39.9%)	131 (51.40%)	258 (42.40%)	8.765	0.012
*Chemotherapy*					
Yes	20 (54.9%)	171 (69.50%)	423 (67.10%)		
No	17 (45.1%)	84 (30.50%)	186 (32.90%)	22.744	< 0.001
*Subtype*					
ER + /HER2−	276 (72.4%)	177 (69.4%)	416 (68.3%)		
ER + /HER2 +	54 (14.2%)	34 (13.3%)	101 (16.6%)	6.514	0.368
HER2 +	15 (3.9%)	11 (4.3%)	35 (5.7%)		
TNBC	36 (9.4%)	33 (12.9%)	57 (9.4%)		
*ER*					
Positive	329 (86.4%)	207 (81.2%)	514 (84.4%)	3.101	0.212
Negative	52 (13.6%)	48 (18.8%)	95 (15.6%)		
*PR*					
Positive	281 (73.8%)	176 (69.0%)	429 (70.4%)	1.97	0.373
Negative	100 (26.2%)	79 (31.0%)	180 (29.6%)		
*HER2*					
Positive	69 (18.1%)	45 (17.6%)	136 (22.3%)	3.786	0.151
Negative	312 (81.9%)	210 (82.4%)	473 (77.7%)		

*ER* estrogen receptor, *PR* progesterone receptor, *Her-2* human epidermal growth factor receptor 2, *IDC* infiltrating ductal carcinoma, *BCS* breast conserving surgery, *TNBC* triple-negative breast cancer

## Discussion

De novo stage IV BC refers to a tumor that has metastasized to other organs of the body with poor prognosis. The therapeutic goal is to improve the OS and the quality of life of patients, and systemic treatment is the first choice. The intervention guideline of local surgery for BC is to relieve symptoms, remove tumor rupture, bleeding, fungal infection and cancer pain without affecting the life of the patient [6].

In our study, the pathology of BC patients with bone metastasis was mostly LuminalA type (ER+/HER2−) (72.5%), which had good prognosis due to the stable endocrine therapy and low proliferative index. Primary site surgical group significantly affected the prognosis of patients (Fig. 1) compared to the non-surgical group (χ^2^ = 146.023, P < 0.001), benefitting most for 70 months in BCS subgroup (Fig. 2) (χ^2^ = 157.117, P < 0.001). The expression of ER, PR, and HER2, histology of invasive ductal carcinoma, married patients, younger age, radiotherapy, and chemotherapy were all protective factors for BC with bone metastasis (P < 0.001). Although the treatment of BC is surgery-based comprehensive treatment, the survival benefit from primary surgery may be related to the following conditions: (1) Surgery could remove the primary tumor site, get rid of the primary tumor cell and tumor stem cells, and reduce the possibility of peripheral release and spread of circulating tumor cells [20–22]; (2) Primary surgery could play an important role at local control of patients: tumor ulcer, infection and other aspects, which improved patients' physical and psychological quality of life; (3) Primary surgery could reduce the burden of tumor and improve the curative effect of tumor chemotherapy [23, 24]; (4) Resection of the primary tumor could reduce the immune suppression of the tumor on the body, activate CD4 and CD8 T lymphocytes, and stimulate the immune response of the body to tumor cells [12, 25, 26].

In the past related studies about advanced invasive carcinoma [23, 27, 28], a phenomenon had been observed in gastric cancer, ovarian cancer, colon cancer that the reduction in tumor burden and an increase in OS were associated, but it was controversial that surgery did not take a survival benefit in de novo stage IV BC [5, 16–18]. However, in recent years, some retrospective studies [7–15] had shown that resection of the primary site of de novo stage IV BC could bring survival benefits, which were most obvious in young patients with positive estrogen receptor, low tumor burden, negative human epidermal growth factor receptor, and bone metastasis only.

We analyzed previous prospective clinical studies. Firstly, the Translational Breast Cancer Research Consortium 013 (TBCRC-013 study) was a prospective multi-institutional registry trial which aimed to evaluate the role of surgery in stage IV breast cancer. Patients diagnosed with stage IV BC at presentation (group A, n = 112) or stage IV within 3 months of diagnosis (group B, n = 16) were enrolled. Early results [29] from this study showed that surgery was associated with improved survival on multivariate analysis (HR 0.28, 95% CI 0.10–0.74, P = 0.01); In addition, 3-year overall survival results showed no difference among patients who responded to first-line therapy [16]. Secondly, the prospective clinical trial was initiated at Tata Memorial Centre in India enrolling 350 patients to receive locoregional treatment (n = 173) or no locoregional treatment (n = 177). The result indicated the surgery could not take survival benefit because of unreasonable systemic therapy. In their study, most patients (92%) with HER2-positive BC did not receive trastuzumab therapy; In addition, they had more metastases ratio (75% vs 25%) while less bone metastases ratio (29%) [17]. At last, the MF07-01 trial conducted by the Turkish Federation seemed to produce positive result. OS was improved for the surgery group at 41.6% as compared to 24.4% in the no surgery group at 5 years. (46 versus 37 months, P = 0.005). Subgroup analysis showed that the survival benefit was associated with ER positive and HER2/neu-negative disease, age under 55, and bone metastases only [18].

Different primary tumor surgery methods took different survival benefit, which might be associated with baseline of patients in terms of T stage, N stage, chemotherapy, radiation therapy. There was lower tumor load, T stage, N stage levels and higher proportion of chemotherapy, radiation therapy in the BCS group, consistent with the fact that the prognosis was better in patients with bone metastasis only from BC with a lower tumor burden. Expanding the scope of surgery and lymph node dissection showed that there was no significant survival benefit [7, 30, 31], consistent with the results of multi-factor analysis in our study, but surgical margin status was correlated with patient prognosis[7]. In addition, Her-2 overexpression was statistically significant in univariate and multivariate analyses (P < 0.05), and Her-2 overexpression might be a protective factor affecting BC bone metastasis, which might be related to anti-Her-2 targeted therapy [32, 33].

Some limitations still exist in this study. Firstly, we are lack of the comprehensive information about systemic treatment, such as endocrine therapy, HER2-targeted therapy, or chemotherapy, which might lead to some bias in the survival analysis. Also, the short of data in the SEER database on events associated with bone metastasis as well as related systematic treatment [34–36] might lead to bias in the conclusions. Another potential issue is the possibility of incomplete or inaccurate claim entry. The tumor burden of patients selected for surgery was relatively low, which was also a part of the surgical bias and might affect the results [37].

## Conclusion

In conclusion, this study shows that primary surgery could improve the prognosis and OS of De novo stage IV BC patients with bone metastasis only. Under the premise of low tumor burden and comprehensive treatment, BCS is a better choice.

## Data Availability

We received permission from the National Cancer Institute, US to access the research data file in the SEER program (Accession number 13705-Nov2019). The datasets analyzed during the current study are available in the SEER repository (SEER*Stat Version 8.3.6) (https://seer.cancer.gov/). The datasets used and/or analysed during the current study are also available from the corresponding author on reasonable request.

## Abbreviations

BC: Breast cancer
TNBC: Triple negative breast cancer
BCS: Breast conserving surgery
SEER: National Cancer Institute surveillance, Epidemiology, and Results Database
OS: Overall survival
AJCC: American Joint Committee on Cancer
ER: Estrogen receptor
PR: Progesterone receptor
Her-2: Human epidermal growth factor receptor 2

## References

[1] Siegel RL, Miller KD, Jemal A (2020). Cancer statistics, 2020. CA Cancer J Clin.

[2] Liede A, Jerzak KJ, Hernandez RK, Wade SW, Sun P, Narod SA (2016). The incidence of bone metastasis after early-stage breast cancer in Canada. Breast Cancer Res Treat.

[3] Jensen A, Jacobsen JB, Nrgaard M, Yong M, Fryzek JP, Srensen HT (2011). Incidence of bone metastases and skeletal-related events in breast cancer patients: a population-based cohort study in Denmark. BMC Cancer..

[4] Filippiadis D, Mavrogenis AF, Mazioti A, Palialexis K, Megaloikonomos PD, Papagelopoulos PJ (2017). Metastatic bone disease from breast cancer: a review of minimally invasive techniques for diagnosis and treatment. Eur J Orthop Surg Traumatol.

[5] CardosoF, Costa A, Senkus E, Aapro M, André F, Barrios CH, *et al*. 3rd ESO-ESMO international consensus guidelines for Advanced Breast Cancer (ABC 3). *The Breast* 2017:244–259. 10.1016/j.breast.2016.10.001 27927580

[6] Gradishar WJ, Anderson BO, Abraham J, Aft R, Agnese D, Allison KH, Blair SL (2020). Breast cancer, Version 3.2020, NCCN Clinical Practice Guidelines in Oncology. J Natl Compr Cancer Netw..

[7] Rapiti E, Verkooijen HM, Vlastos G, Fioretta G, Neyroud-Caspar I, Sappino AP (2006). Complete excision of primary breast tumor improves survival of patients with metastatic breast cancer at diagnosis. J Clin Oncol.

[8] Gnerlich J, Jeffe DB, Deshpande AD, Beers C, Zander C, Margenthaler JA (2007). Surgical removal of the primary tumor increases overall survival in patients with metastatic breast cancer: analysis of the 1988–2003 SEER data. Ann Surg Oncol.

[9] Rao R, Feng L, Kuerer HM, Singletary SE, Bedrosian I, Hunt KK (2008). Timing of surgical intervention for the intact primary in stage IV breast cancer patients. Ann Surg Oncol.

[10] Bafford AC, Burstein HJ, Barkley CR, Smith BL, Lipsitz S, Iglehart JD (2009). Breast surgery in stage IV breast cancer: impact of staging and patient selection on overall survival. Breast Cancer Res Treat.

[11] Harris E, Barry M, Kell MR (2013). Meta-analysis to determine if surgical resection of the primary tumour in the setting of stage IV breast cancer impacts on survival. Ann Surg Oncol.

[12] Rashid O, Nagahashi M, Ramachandran S, Graham L, Yamada A, Spiegel S (2013). Resection of the primary tumor improves survival in metastatic breast cancer by reducing overall tumor burden. Surgery.

[13] AlJohani B, AlMalik O, Anwar E, Tulbah A, Alshabanah M, AlSyaed A (2016). Impact of surgery on survival in stage IV breast cancer. Breast J.

[14] Thomas A, Khan SA, Chrischilles EA, Schroeder MC (2016). Initial surgery and survival in stage IV breast cancer in the United States, 1988–2011. JAMA Surg.

[15] Vohra NA, Brinkley J, Kachare S, Muzaffar M (2018). Primary tumor resection in metastatic breast cancer: a propensity-matched analysis, 1988–2011 SEER data base. Breast J.

[16] King TA, Lyman J, Gonen M, Reyes S, Hwang ESS, Rugo HS, Liu MC (2016). A prospective analysis of surgery and survival in stage IV breast cancer (TBCRC 013). J Clin Oncol.

[17] Badwe R, Hawaldar R, Nair N, Kaushik R, Parmar V, Siddique S (2015). Locoregional treatment versus no treatment of the primary tumour in metastatic breast cancer: an open-label randomised controlled trial. Lancet Oncol.

[18] Soran A, Ozmen V, Ozbas S, Karanlik H, Muslumanoglu M, Igci A (2018). Randomized trial comparing resection of primary tumor with no surgery in stage IV breast cancer at presentation: protocol MF07-01. Ann Surg Oncol.

[19] Edge SB, Compton CC (2010). The American Joint Committee on Cancer: the 7^th^ edition of the AJCC cancer staging manual and the future of TNM. Ann Surg Oncol.

[20] Cristofanilli M, Budd GT, Ellis MJ, Stopeck A, Matera J, Miller MC (2004). Circulating tumor cells, disease progression, and survival in metastatic breast cancer. N Engl J Med.

[21] Algizawy SM, Essa HH, El-Gezawy E, Omar NN, Sayed DM (2016). Circulating tumor cells as an early predictive marker of disease progression in metastatic breast cancer patients. Cancer Biol.

[22] Lang JE, Babiera GV (2007). Locoregional resection in stage IV breast cancer: tumor biology, molecular and clinical perspectives. Surg Clin North Am.

[23] Griffiths CT, Parker LM, Lee S, Finkler NJ (2002). The effect of residual mass size on response to chemotherapy after surgical cytoreduction for advanced ovarian cancer: long-term results. Int J Gynecol Cancer.

[24] Dauplat J, Boudec GL, Pomel C, Scherer C (2000). Cytoreductive surgery for advanced stages of ovarian cancer. Semin Surg Oncol.

[25] Danna EA, Sinha P, Gilbert M, Clements VK, Pulaski BA, Ostrand-Rosenberg S (2004). Surgical removal of primary tumor reverses tumor-induced immunosuppression despite the presence of metastatic disease. Cancer Res.

[26] Rashid OM, Nagahashi M, Ramachandran S, Milstien S, Spiegel S, Takabe K. Resection of primary tumor improves survival in mouse metastatic breast cancer model. Cancer Res. 2010; 70(8) ISSN: 0008-5472.

[27] Hallissey MT, Allum WH, Roginski C, Fielding JW (1988). Palliative surgery for gastric cancer. Cancer.

[28] Martin R, Paty P, Fong Y, Grace A, Cohen A, DeMatteo R (2003). Simultaneous liver and colorectal resections are safe for synchronous colorectal liver metastasis. J Am Coll Surg.

[29] King T, Lyman J, Gonen M, Voci A, Boafo C, Hwang E, et al. TBCRC 013: a prospective analysis of the role of surgery in stage IV breast cancer. Cancer Res 2013;73(24 Suppl): Abstract nr P2-18-09. 10.1158/0008-5472.SABCS13-P2-18-09.

[30] Khan SAM, Stewart AKM, Morrow MM (2002). Does aggressive local therapy improve survival in metastatic breast cancer?. Surgery.

[31] McGuire KP, Eisen S, Rodriguez A, Meade T, Cox CE, Khakpour N (2009). Factors associated with improved outcome after surgery in metastatic breast cancer patients. Am J Surg.

[32] Liao N (2016). HER2-positive breast cancer, how far away from the cure?-on the current situation of anti-HER2 therapy in breast cancer treatment and survival of patients. Chin Clin Oncol.

[33] Lv S, Wang Y, Sun T, Wan D, Sheng L, Li W (2018). Overall survival benefit from trastuzumab-based treatment in HER2-Positive metastatic breast cancer: a retrospective analysis. Oncol Res Treat.

[34] Fornetti J, Welm AL, Stewart SA (2018). Understanding the bone in cancer metastasis. J Bone Miner Res.

[35] Yanae M, Fujimoto S, Tane K, Tanioka M, Fujiwara K, Tsubaki M (2017). Increased risk of SSEs in bone-only metastatic breast cancer patients treated with zoledronic acid. J Bone Oncol.

[36] Pulido C, Vendrell I, Ferreira AR, Casimiro S, Mansinho A, Alho I, Costa L (2017). Bone metastasis risk factors in breast cancer. Ecancermedicalscience.

[37] Olson JA, Marcom PK (2008). Benefit or bias? The role of surgery to remove the primary tumor in patients with metastatic breast cancer. Ann Surg.

